# Neuroscience Literacy and Academic Outcomes: Insights from a University Student Population

**DOI:** 10.3390/brainsci15010044

**Published:** 2025-01-04

**Authors:** Abeer F. Almarzouki, Arzan I. Alqahtani, Jumana K. Baessa, Dhuha K. Badaood, Rwdyn R. Nujoom, Raneem W. Malibari, Elaf M. Aljared, Reema S. Alghamdi

**Affiliations:** 1 Department of Clinical Physiology, Faculty of Medicine, King Abdulaziz University, Abdullah Al-Sulaiman Street, Al Jamiaa District 80200, Jeddah 21589, Saudi Arabia; 2 Faculty of Medicine, King Abdulaziz University, Jeddah 21589, Saudi Arabia

**Keywords:** neuroscience literacy, neuroliteracy, neuromyth, educational neuroscience, study habits, learning styles, academic outcomes

## Abstract

**Background/Objectives:** There is growing interest in neuroscience-informed education, as well as neuroscience-derived strategies that maximise learning. Studies on neuroscience literacy and neuromyths, i.e., understandings or misconceptions about the brain, have primarily focused on their prevalence in educators, and few studies have examined their impact on students’ study habits or academic performance. **Methods:** To address this gap, we surveyed 576 university students in different academic programmes to investigate the relationship between neuromyths and academic outcomes in university students. In this quantitative, cross-sectional study design, we used a validated neuroscience knowledge survey and the Revised Two-factor Study Process (R-SPQ-2F) Questionnaire. We also inquired about students’ interest in, exposure to, and awareness of neuroscience, as well as their academic grades. **Results:** Students showed significant awareness of and interest in neuroscience; this was highest among students in health science programmes and lowest among students in computer and engineering programmes. The most common sources of general neuroscience knowledge were internet articles. Higher neuroscience literacy was associated with higher interest in neuroscience and having taken more neuroscience courses. Neuromyth scores were also better among those with higher neuroscience literacy scores. Higher neuroscience literacy scores were significantly associated with higher grades, higher surface strategy scores, and lower surface motive study habits. **Conclusions:** Our study sheds light on the variations in foundational neuroscience literacy among students in different academic programmes. It also provides insight into how this foundation affects academic performance and study habits. This insight may help guide educational policymakers to adopt neuroscience-based strategies that may be beneficial for learning.

## 1. Introduction

Over the last few decades, educators and researchers have shown increasing interest in applying neuroscience to education [1,2]. Despite advances in this area [3], real-world applications of neuroscience literacy, defined as “understanding the brain and how it functions” [4], in educational settings are lacking [5]. Dekker et al. first introduced the concept of neuroscience literacy in their investigation of trainee teachers in the United Kingdom, concluding that this group showed an alarming number of misconceptions [6]. Neuroscience literacy may improve education by facilitating an understanding of the brain mechanisms that drive learning and integrating this knowledge into educational systems [7]. Nevertheless, a recent systematic review of 24 studies, involving 13,767 educators in nearly 20 different countries, indicated that a significant gap remains between neuroscience knowledge and education [8].

Neuromyths, which are incorrect beliefs about the brain and neuroscience, are widespread [9]. A recent study in Quebec found that 72.6% of the surveyed teachers reported using the hemispheric dominance neuromyth [10], which suggests that students with left-brain dominance are more calculative and logical, whereas right-brain learners are more artistic and creative [11]. In addition, 97.6% of the teachers surveyed mentioned employing the “learning styles” neuromyth with their students [10]. This neuromyth is quite popular among educators, who might group students according to their supposed best learning style (e.g., auditory or visual learning style), despite its questionable efficacy in improving education outcomes [12].

Most studies in the context of neuroscience literacy or neuromyths have looked at their prevalence and beliefs in teachers, prospective teachers [13,14,15,16], or specific student populations [17,18,19]. For example, a small (*n* = 12) study of high school students found that even if students believe they fit a particular learning style, they do not necessarily learn better when using it [20]. Few studies have examined how diverse academic or educational backgrounds among students influence neuroscience literacy or neuromyths [14]. Students in health science programmes are more exposed to neuroscience courses and, hence, may show more neuroscience foundation knowledge compared with those who have not been exposed to neuroscience programmes [21]. That said, even fewer studies have investigated whether neuroscience literacy can predict academic grades or influence study habits [14]. Studies generally suggest that university students are not immune to misconceptions about neuroscience, although it has been hinted that this could differ according to the academic programme [14,18]. Furthermore, while neuroscience-based education has primarily been a focus for health-related science programmes [19,22], there is growing interest in integrating and applying educational neuroscience in other academic fields. However, thus far, the extent and variety of this integration have not been thoroughly explored [23]. Thus, to better examine the role of neuroscience literacy in learning, and whether understanding the brain’s learning mechanisms can benefit education, further studies on the application of neuroscience literacy in the context of different student populations are needed.

In this study, we investigated neuroscience literacy levels among university students from different academic programmes. In addition, given the lack of research connecting neuroscience literacy to academic outcomes in students, we examined the impact of neuroscience knowledge on study habits and academic achievement among university students. This study provides a unique contribution by studying neuroscience literacy in university students, contrasting with the existing focus on educators. This study aimed to answer the following questions: How does neuroscience literacy differ among university students in different academic programmes? Do those with higher neuroscience literacy have a stronger academic performance? Does neuroscience literacy influence study habits among this student population?

## 2. Materials and Methods

### 2.1. Study Area and Population

This study was conducted during the 2023 academic year. The study population consisted of students enrolled in King Abdulaziz University, who were invited to participate through online advertisements. Those who responded were screened by phone for eligibility and invited to participate if they met the eligibility criteria. Individuals were eligible to participate if they were (a) students actively enrolled at a university, (b) over 18 years of age, and (c) able to understand and speak English. We excluded students who (a) were in special education, (b) had intellectual or sensory disabilities, and (c) were studying in non-traditional classrooms. By focusing on this population using these eligibility criteria, we aimed to establish a more uniform educational setting to test our main hypotheses.

Ethical approval was obtained from the local ethical committee at King Abdulaziz University (Reference No. 206-23). All participants were required to read an information page and sign a consent form before agreeing to participate in the study. All information obtained from the participants was kept anonymous and securely stored.

### 2.2. Data Collection Instruments

Using an online data collection form that was generated in Google Forms and distributed through social media platforms, participants were asked to provide basic demographic information (date of birth, sex, marital status, and work status) and their current academic program. They were also asked about their general aptitude (GAT) (maximum score = 100), summative assessment (maximum score = 100), and cumulative GPA scores (highest possible grade point average = 5). Information on their previous education was also collected, including whether they were enrolled in local or international high school programmes or gifted education programmes. Participants were also asked to rate their level of interest in neurology, neuroscience, human behaviour, and psychology on a 5-point Likert scale ranging from “Strongly agree” to “Strongly disagree”; the former suggests a higher interest in these topics, while the latter indicates less interest. Participants were inquired about the number of courses specifically focused on neurology, neuroscience, human behaviour, or psychology they had previously completed, regardless of the level of study (e.g., undergraduate or postgraduate modules). Lastly, they were asked to identify the sources they had used to obtain information or improve their knowledge in these areas. The sources mentioned included educational curricula or training materials such as textbooks and classroom lectures, books, internet articles, YouTube videos, Google searches, scientific journals, published peer-reviewed articles, and public media such as television programmes or documentaries.

### 2.3. Neuroscience Literacy

Neuroscience literacy was assessed using the neuroscience knowledge survey previously created and validated by Im et al. [19]. This scale measures participants’ knowledge and understanding of various aspects of neuroscience and is one of the only validated instruments for this purpose. The survey also assessed misconceptions about neuroscience and beliefs in neuromyths. This consisted of 59 true or false statements organised into six sections, each representing a different area of interest, including general knowledge about the brain (14 statements), brain function (8 statements), brain development (10 statements), brain structure (12 statements), neuroimaging (6 statements), and the application of neuroscience findings (9 statements). Neuromyth questions comprised any questions where the correct answer was false (e.g., Brain development has finished by the time children reach secondary school), thus, in addition to scores in each section, participants had a neuromyth score, which was the number of correct responses to these 31 questions. Participants were asked to indicate their agreement with each of the 59 statements by selecting “Yes”, “No”, or “I don’t know”. A high score in any area indicated more accurate knowledge, while a high grade in neuromyth questions indicated more accurate discrimination of myths. Neuromyth scores were analysed as a separate category from each survey section. The scoring and data analysis of the survey were based on the work by Im et al. [19].

### 2.4. Study Habits

The Revised Two-factor Study Process (R-SPQ-2F) Questionnaire is a validated 20-item self-report tool designed to evaluate students’ study habits [24], which addresses our research questions related to study habits. The questionnaire categorises students into two groups based on their approaches to learning: deep or surface approaches. Each approach is further divided into two subscales: motive and strategy. The deep approach subscales measure the extent to which students are driven by intrinsic factors. The deep motive scale assesses curiosity as a motivating factor, while the deep strategy scale evaluates the level of effort students are willing to invest in acquiring a comprehensive understanding of the material. The surface approach scale examines the degree to which students are driven by extrinsic factors. The surface motive scale assesses motivation by fear of failure and the desire to complete tasks with minimal effort. The surface strategy subscale provides insights into the extent to which students rely on memorisation and narrowly focused learning techniques. The questionnaire includes five statements for each study habit type—(1) deep motive, (2) deep strategy, (3) surface motive, and (4) surface strategy—each answered on a 5-point Likert scale for a maximum of 25 points in each category. Participants were asked to indicate their agreement with each statement by selecting from five options: this item is never or only rarely true of me, this item is sometimes true of me, this item is true of me about half the time, this item is frequently true of me, and this item is always or almost always true of me. Total “deep” and “surface” scores were calculated by adding the two categories together for a maximum of 50 points in each. As a result, each student obtained surface motive, surface strategy, deep motive, deep strategy, total surface, and total deep scores reflecting these attributes. The scoring and data analysis of the survey were based on the original published work [24].

### 2.5. Data Analysis

The descriptive statistics included means and standard deviations reported for continuous numbers (e.g., age) and counts with relative proportions for categorical variables (e.g., marital status). Outcome measures beyond three standard deviations of their mean were removed as outliers. The differences in outcomes (e.g., study habits, neuroscience literacy scores, and GPA) among students in different programmes were tested using linear regression modelling. Neither age, sex, nor year of study significantly improved the model fit (log-likelihood) when comparing the study habits or literacy scores of students in different programmes. However, all three significantly improved the comparisons of GPA and were controlled as covariates. Model residuals were confirmed to be normally distributed using the Kolmogorov–Smirnov test. Multicollinearity was tested among all predictors by calculating the variance inflation factor (VIF), which was negligible (all VIFs < 2.0). The academic programmes were grouped into four broad categories: (1) humanities (art and design, business administration, tourism, languages and humanities, religious studies, law and political sciences, media and communication, and public administration), (2) natural sciences (science programmes including biology, chemistry, physics, and environmental sciences), (3) engineering and computer sciences (computer and information sciences, engineering, electrical engineering, and architecture), and (4) health sciences (nursing, dentistry, applied medical sciences, rehabilitation sciences, and medicine). Students in their foundation year were excluded.

## 3. Results

### 3.1. Study Sample

This study included 576 participants (35.9% male) with a mean age of 21.7 years. A detailed breakdown of participant characteristics (demographic information and academic programme) is provided in Table 1. Most participants were single (96.4%). The vast majority were in-person students (96.4%) as opposed to those studying online or through other modes. About half of the participants in our sample were medical students (45.3%). Participants were predominantly in their third year of study (47.0%), and three-quarters were graduates of local high school programmes (75.7%). Most participants had high academic performance, scoring more than 85% on average on all three academic performance tests (Table 1). The majority of participants were either in health sciences, natural sciences, engineering and computer sciences, or humanities programmes, with a small minority (4.2%, n = 24) in other programmes. Participants in other programmes were excluded from analyses of programme differences.

Table 2 describes the overall neuroscience awareness, interest, and exposure, as well as the neuroscience literacy scores. Most participants (82.1%) reported being aware of neuroscience. The neuroscience literacy score was 21.82 (out of 59), and the neuromyth score was 8.33 (out of 31). While 64.8% reported not having taken any neuroscience courses, more than half either strongly agreed (28.0%) or agreed (37.3%) that they were interested in the topic. The most common sources of general neuroscience knowledge were reported to be internet articles, YouTube videos, Google searches, or other websites (71.2%). Other sources were less common, including books (30.7%) or peer-reviewed journal articles (17.9%). Neuromyth scores were significantly associated with neuroscience literacy scores (*r* = 0.762, *p* < 0.001), showing that those with a better overall understanding of neuroscience also scored better on questions pertaining to neuromyths, i.e., were better able to dismiss neuromyth statements. This correlation held for students in health science programmes (*r* = 0.719, *p* < 0.001), natural sciences (*r* = 0.765, *p* < 0.001), engineering and computer sciences (*r* = 0.802, *p* < 0.001), and humanities (*r* = 0.741, *p* < 0.001).

### 3.2. Academic Programmes and Neuroscience Literacy

**Figure 1:**
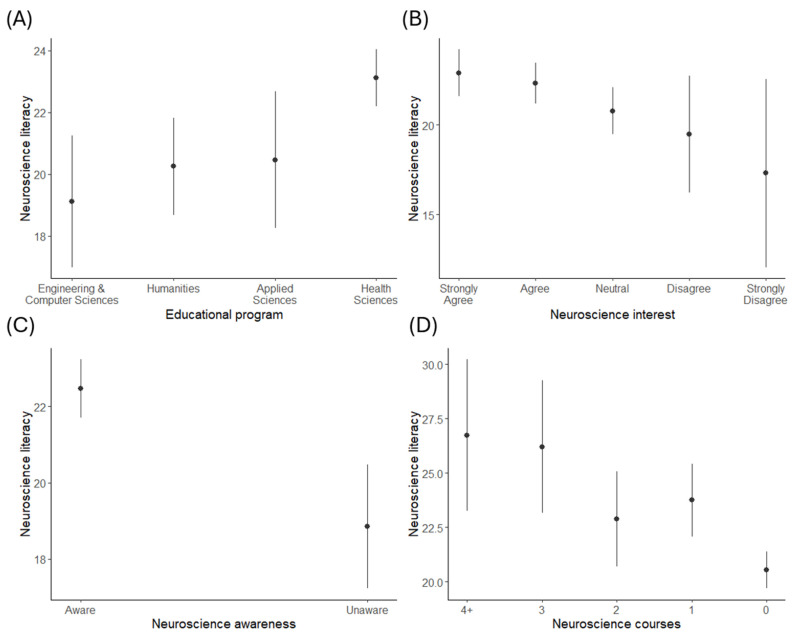
Associations between neuroscience literacy scores and academic programmes (**A**), interest in neuroscience (**B**), awareness of neuroscience (**C**), and the number of neuroscience courses taken (**D**).

Neuroscience literacy scores, awareness, interest, and exposure are broken down by academic programme in Table 3. Students in health science programmes reported the highest awareness of neuroscience (91%) and obtained the highest neuroscience literacy scores. Students in natural science programmes had taken more neuroscience courses and demonstrated a similar level of interest in the topic, resulting in the second-highest neuroscience literacy scores. Conversely, the lowest awareness and neuroscience literacy scores in all groups were seen for students in engineering and computer science programmes (65% and 19.12, respectively; see Figure 1A).

### 3.3. Interest in Neuroscience and Neuroscience Literacy

The association between neuroscience literacy and an interest in neuroscience is shown in Figure 1B. Participants who reported a greater interest in neuroscience scored significantly higher on the neuroscience literacy scale (*F*(4,571) = 2.69, *p* = 0.030). Neuroscience literacy scores were lowest in those who strongly disagreed that they were interested in neuroscience (17.3), followed by those who disagreed (19.5), were neutral (20.8), agreed (22.3), or strongly agreed (22.9). Participants in health or natural science programmes were more likely to agree or strongly agree that they had an interest in neuroscience (29.9% and 35.7%, respectively) compared to those in engineering and computer sciences (21.7%) or humanities (22.3%). This difference in the level of interest among students in different academic programmes was statistically significant (χ^2^ = 24.27, *p* = 0.019).

### 3.4. Awareness of Neuroscience and Neuroscience Literacy

Differences in awareness between programmes were also reflected in the neuroscience literacy scores, as shown in Figure 1C. Neuroscience literacy was significantly higher in students who self-identified as being aware of neuroscience (*t*(574) = 3.96, *p* < 0.001). Neuroscience literacy scores were highest in students enrolled in health science programmes (23.1, *SE* = 0.47), followed by those in natural sciences (20.5, *SE* = 1.13) and humanities (20.2, *SE* = 0.79), with students in engineering and computer science programmes receiving the lowest scores (19.1, *SE* =1.09). These differences were statistically significant (*F*(3,548) = 6.37, *p* < 0.001). Post hoc testing showed that scores were significantly lower for students in engineering and computer sciences (*t*(548) = 3.37, *p* = 0.005) and humanities (*t*(548) = 3.09, *p* = 0.011) compared to those in health science programmes.

### 3.5. Exposure to Neuroscience Scores and Neuroscience Literacy

Neuroscience literacy was significantly higher in students who had taken more neuroscience courses (*t*(574) = 5.15, *p* < 0.001). It was also significantly higher among those who reported receiving neuroscience information through their educational curriculum or training (*t*(574) = 2.13, *p* = 0.033), peer-reviewed journal articles (*t*(574) = 2.23, *p* = 0.026), or internet articles (*t*(574) = 2.16, *p* = 0.032). However, neuroscience literacy was not significantly higher among those who relied on books or public media than those who did not (see Figure 1D).

### 3.6. Study Habits

The mean and standard deviations of surface or deep motive/strategy scores across programmes with more than 20 students are shown in Table 4. Overall surface study scores did not significantly differ among students in different academic programmes, though differences were seen in surface motive and surface strategy study scores (Table 4). Surface motive study scores were highest among students in engineering and computer sciences, while surface strategy study scores were highest among students in health sciences, with students in engineering and computer sciences ranking second. Deep study scores did not significantly differ among students in different academic programmes, either overall or in terms of motive and strategy scores.

### 3.7. Academic Performance

GPA was highest in students enrolled in health science programmes (4.39, *SD* = 0.45), followed by those in engineering and computer sciences (4.31, *SD* = 0.58) and humanities (4.19, *SD* = 0.64). It was lowest in students enrolled in natural science programmes (4.09, *SD* = 0.71). In addition, GPA was significantly higher in younger students (*t*(532) = −4.32, *p* < 0.001), women (*t*(532) = 5.11, *p* < 0.001), and those in later years of study (*t*(532) = 3.41, *p* < 0.001), when controlling for each of these factors. Overall differences in GPA among students in different programmes were significant after adjusting for age, sex, and year of study (*F*(3,532) = 5.09, *p* < 0.001).

GPA was significantly positively related to both summative assessment scores (*r* = 0.443, *p* < 0.001) and general aptitude scores (*r* = 0.377, *p* < 0.001). In addition, both summative assessment scores (*F*(3,548) = 51.76, *p* < 0.001) and general aptitude scores (*F*(3,548) = 45.16, *p* < 0.001) were sensitive to the same differences between academic programmes as GPA. For brevity, GPA was used in subsequent analyses.

### 3.8. Interplay Between Study Habits, Neuroscience Literacy, and Academic Performance

**Figure 2:**
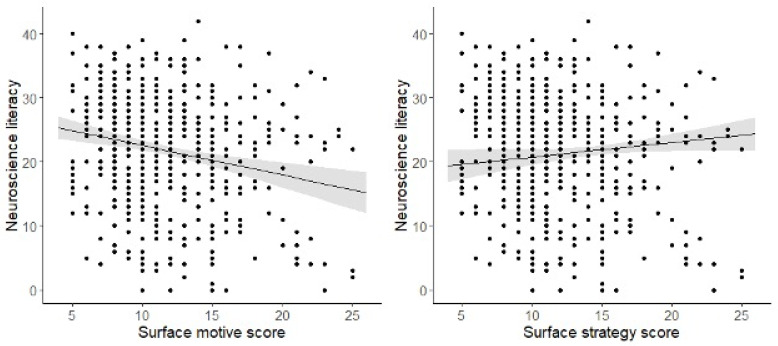
Significant associations between neuroscience literacy scores and surface motive/strategy study habit scores.

Students enrolled in engineering and computer science programmes had the highest surface motive scores (Table 3) but the least exposure to neuroscience. Conversely, students in health science programmes had the highest surface strategy study scores and the most exposure to neuroscience. We tested whether neuroscience literacy was associated with any of the four study domain scores (surface or deep; motive or strategy). Those with higher neuroscience literacy had significantly lower surface motive study scores (*t*(565) = 3.89, *p* < 0.001) and significantly higher surface strategy study scores (*t*(565) = 2.03, *p* = 0.043). Notably, this pattern aligns with the results for students in health science programmes. The associations of neuroscience literacy with surface motive and surface strategy scores are shown in Figure 2.

While only 65% of students in engineering and computer science programmes reported a general awareness of neuroscience, the proportion was 91% among students in health science programmes. Furthermore, those who reported higher awareness of neuroscience had significantly higher neuroscience literacy scores (*t*(574) = 3.96, *p* < 0.001). Therefore, while study scores are related to neuroscience literacy, differences in this regard may stem from differing levels of exposure across programmes. Alternatively, the correlation between neuroscience literacy and study scores could be due to programme-specific differences in the latter. However, a direct causal association would require further study. Neuroscience literacy was not significantly associated with deep motive or deep strategy study scores. We tested whether the effect of either surface motive or surface strategy study habit scores on neuroscience literacy was moderated by academic programmes. However, the interactions were non-significant in both cases. This could be due to the smaller sample sizes in each academic programme compared with the full sample, resulting in less sensitivity for detecting variations in their slopes.

Additionally, we tested whether overall GPA was associated with study habits in any of the four domains using this same procedure but adjusting for age, sex, and year of study. GPA was not significantly associated with surface motive or surface strategy study scores, suggesting that the associations between neuroscience literacy and study habits in these domains are unconnected to academic performance. However, GPA was significantly higher in students with higher deep motive strategy scores (*t*(549) = 2.17, *p* = 0.031). There was no statistically significant variation in these patterns between programmes (i.e., there was no significant interaction between study habits and programmes). Notably, deep motive strategy scores did not significantly differ among students in different academic programmes (Table 3).

Lastly, we tested for associations between GPA and neuroscience literacy scores, adjusting for age, sex, and year of study. Higher neuroscience literacy scores were significantly associated with higher GPA (*t*(566) = 2.51, *p* = 0.012), as well as higher summative assessment scores (*t*(571) = 2.73, *p* = 0.006). While the positive association with GPA was only significant among students in natural science programmes (*t*(536) = 3.79, *p* < 0.001), the association with summative assessment score was uniformly positive and did not differ between programmes (F(3,541) = 0.283, *p* = 0.838). When adjusting for the four study habit domains, the association with GPA became non-significant (*t*(567) = 2.58, *p =* 0.053), while the association with the summative assessment score remained significant (*t*(567) = 2.58, *p* = 0.010).

## 4. Discussion

Despite increasing interest, the implementation of educational neuroscience in real-world practice remains challenging. Although the negative impact of beliefs in neuromyths in education is still under investigation [25], such beliefs may lead individuals to invest in inefficient ways of learning. A significant aspect of this challenge is the lack of research on the impact of such beliefs in real-world learning settings, especially with respect to students, despite their role as the recipients of learning. Importantly, the lack of research on students means that there is a lack of information on the target of direct neuroliteracy interventions; to design better interventions, more research on students is needed.

Overall, our student population reported a high awareness of and interest in neuroscience; this aligns with the overall growing interest in neuroscience [3]. The levels of awareness and interest in neuroscience differed among students in different academic programmes, but even in the cohort of students with the lowest exposure to neuroscience (i.e., computer science and engineering students), most showed a high awareness of neuroscience and either strongly agreed or agreed that they had an interest in it. The students with the highest awareness of and interest in neuroscience were those in health and natural science programmes; these finding were similar to those reported in previous studies [14]. It could be that students who are inherently interested in neuroscience voluntarily enrol in either health or natural science programmes—or students already enrolled in these programmes—are more likely to be exposed to concepts related to neuroscience and develop an interest.

Interestingly, only a small percentage of the students in our sample gained their general knowledge of neuroscience from formal education, with most referring to the internet. This finding confirms the importance of internet-based resources in gaining knowledge and education among the younger population [26]. Students who showed an interest in neuroscience naturally obtained the highest neuroscience literacy scores. This highlights the importance of promoting student interest to boost learning and advance education [27]. Neuroscience literacy scores were also positively associated with the number of neuroscience courses students had taken and the presence of the topic in their educational curriculum. Since they took neuroscience-based courses as part of their programmes, this may further explain why students in health or natural science programmes scored higher in neuroscience literacy compared with others. A previous study on neuromyths investigated the prevalence and predictors of these beliefs in three target populations [9]: educators, those who have been exposed to neuroscience, and the public. Educators and those with high levels of exposure to neuroscience believed in fewer neuromyths [9]. This further supports previous calls to incorporate neuroscience-based courses into students’ educational curricula [28].

The application of neuroscience-based research to real-life educational practices was discussed by Pincham et al. [29], who suggested a four-stage model of neuroscience implementation to empower education [29]. However, little is known about how such knowledge translates into real-life student learning practices. In our study, we found that neuroscience literacy scores were associated with a lower surface motivation to study, that is, students with higher neuroscience literacy scores are more driven by intrinsic rather than extrinsic motivation. One might conclude that a better surface strategy score would lend itself to higher neuroscience literacy or vice versa. However, this pattern may actually describe the study habits of students in health science programmes, who represent the bulk of our sample (56%). Students in health science programmes had lower surface motive and higher surface strategy scores than students in other academic programmes. These students were also more exposed to neuroscience courses. Thus, rather than neuroscience literacy driving study habits, or vice versa, it may be the programme that dictates studying behaviours and neuroscience literacy scores. The type, design, and level of the course taught, including instructor-led cues, all inform students’ study habits [30]. Further research is necessary to determine the directionality and causality of these findings. Interestingly, students in health science programmes had the highest academic scores among all the academic programmes, as well as the highest intrinsic motivation scores, indicating that intrinsic motivation may be more effective than extrinsic motivation in promoting better academic performance [30].

Finally, our study tested the association between neuroscience literacy and academic performance. Higher neuroscience literacy scores were associated with higher overall GPA and summative assessment scores. Summative assessments are more broadly focused on knowledge that is applicable across disciplines [31], and students with higher summative scores may be more knowledgeable overall, including in neuroscience. Accordingly, we cannot draw causal inferences because of uncontrolled confounding factors, such as self-selection into disciplines that value neuroscience education to various degrees. The inter-programme GPA variations associated with instructional differences rather than individual differences also require clarification. Understanding predisposing factors can guide evidence-based interventions to reduce the disparities in foundational neuroscience comprehension that contribute to gaps in achievement. A more effective method for clarifying this ambiguity would be to design an interventional study that starts with a homogeneous group and compares the results of students before and after taking a neuroscience-based course.

### Study Limitations

Although this research provides valuable initial insights into the level of neuroscience literacy among university students in various fields of study, as well as its impact on study habits and academic performance, certain limitations must be acknowledged. Firstly, about half of our sample comprised medical students in their third year, which is when neuroscience courses are incorporated into their curriculum. This may have resulted in oversampling students with higher awareness of and exposure to neuroscience. More studies comparing first-year students with those in later years could help clarify how study habits change over time [32]. There were similarly unbalanced sample sizes across programmes, with as few as 56 in natural sciences and 324 in health sciences.

Secondly, the cross-sectional design assessed neuroscience literacy at a single time point rather than longitudinally. Thirdly, the data obtained relied on self-reporting for variables such as interest levels and study habits, thereby introducing a risk of social desirability bias. Recall bias, inaccurate or duplicate responses cannot be excluded in online surveys making it difficult to establish causality in this study.

Another limitation is that students were not directly asked if they used neuroscience-driven techniques for their study habits; rather, this was inferred from the significant association between study habits and neuroscience literacy scores. As such, this could be explored in future research. Furthermore, the non-experimental nature of this study limits our ability to make causal claims, as unmeasured confounders may influence the observed associations. This study used a convenience sampling method that was limited to a single university population, which may be justified by the exploratory nature of the study but must be strengthened in future work by using a more rigorous sampling method and recruiting a more representative sample, including greater sampling of students in underrepresented programmes. In addition, this study relied on self-reported understandings of complex concepts rather than objective testing for knowledge, which may result in individuals misrepresenting their level of knowledge in certain areas.

Targeted longitudinal investigations with more balanced disciplinary representation and the use of direct academic metrics could strengthen the inferences regarding the effects of neuroscience literacy. Additionally, incorporating qualitative methodologies could further clarify self-reported tendencies and provide richer insight into academic performance.

## 5. Conclusions

This is one of the first studies to explore the prevalence, application, and impact of neuroscience literacy in a student population. We found high interest and awareness of neuroscience among our student population, especially students in health science programmes. Those with higher neuroscience literacy scores had higher grades, higher deep study motive scores, and more surface strategy study habits. This study provides insights into the discrepancies in foundational neuroscience literacy among students in different academic programmes. Future studies could longitudinally assess targeted interventions and examine objective metrics.

Although neuroscience-based education can help bridge the gap between science and its applications, understanding the differences in foundational knowledge can help optimise education by integrating neuroscience-literacy-boosting strategies. If interest in neuroscience indeed promotes educational success, exploring methods of promoting student interest in the field can help optimise education and learning.

## Figures and Tables

**Table 1:** Participant demographic and academic characteristics (*n* = 576).

Characteristics	*n* (%)
Age, mean (SD)	21.73 (2.05)
Sex (Male)	207 (35.9)
Study year	
First	61 (10.6)
Second	72 (12.5)
Third	271 (47)
Fourth	107 (18.6)
Fifth	41 (7.1)
Sixth	11 (1.9)
Seventh	13 (2.3)
Academic programme	
Health sciences	324 (56.3)
Natural sciences	56 (9.7)
Engineering and computer science	60 (10.4)
Humanities	112 (19.4)
Other	24 (4.2)
Academic performance, mean (SD)	
General aptitude score (GAT)	85.21 (7.49)
Summative assessment score	84.78 (9.32)
Grade point average (GPA)	4.31 (0.55)

**Table 2:** Participants’ study habits and neuroscience exposure (*n* = 576).

Characteristics	*n* (%)
Aware of neuroscience (Yes)	473 (82.1)
Neuroscience literacy score, mean (SD)	21.82 (8.50)
Neuroscience myth score, mean (SD)	8.33 (4.21)
Interested in neuroscience	
Strongly agree	161 (28)
Agree	215 (37.3)
Neutral	164 (28.5)
Disagree	26 (4.5)
Strongly disagree	10 (1.7)
Neuroscience courses	
None	373 (64.8)
One	96 (16.7)
Two	56 (9.7)
Three	29 (5)
Four or more	22 (3.8)
Neuroscience information sources	
Internet articles, YouTube, Google, and other	410 (71.2)
Education curriculum or training	262 (45.5)
Public media	189 (32.8)
Books	177 (30.7)
Peer-reviewed journals	103 (17.9)

**Table 3:** Interest in and exposure to neuroscience materials by academic programme (*n* = 552). The significance of group differences was calculated using linear regression and chi-squared testing, with significance shown using asterisks (*, *p* < 0.05).

Characteristics	Health Sciences, *n* (%) (*n* = 324)	Natural Sciences, *n* (%) (*n* = 56)	Humanities, *n* (%) (*n* = 112)	Engineering and Computer Sciences, *n* (%) (*n* = 60)	Group Differences (*n* = 552)	*p*-Value
Aware of neuroscience	296 (91.4)	44 (78.6)	77 (68.8)	39 (65)	χ2 (3, n = 552) = 45.82	0.001 *
Neuroliteracy, mean (SD)	23.12 (8.40)	20.46 (9.22)	20.25 (7.87)	19.12 (8.99)	F (3, 548) = 6.37	0.001 *
Neuromyth, mean (SD)	8.90 (4.30)	7.16 (3.85)	8.24 (4.00)	7.02 (4.23)	F (3, 548) = 5.41	0.001 *
Interest in neuroscience						
Strongly agree	97 (29.9)	20 (35.7)	25 (22.3)	13 (21.7)	χ2 (12, n = 522) = 24.27	0.019 *
Agree	133 (41)	20 (35.7)	33 (29.5)	21 (35)		
Neutral	79 (24.4)	14 (25)	47 (42)	18 (30)		
Disagree	11 (3.4)	1 (1.8)	6 (5.4)	6 (10)		
Strongly disagree	4 (1.2)	1 (1.8)	1 (0.9)	2 (3.3)		
Neuroscience courses						
None	201 (62)	33 (58.9)	73 (65.2)	46 (76.7)	χ2 (12, n = 522) = 15.54	0.213
One	60 (18.5)	9 (16.1)	20 (17.9)	5 (8.3)		
Two	29 (9)	6 (10.7)	14 (12.5)	7 (11.7)		
Three	20 (6.2)	3 (5.4)	3 (2.7)	1 (1.7)		
Four or more	14 (4.3)	5 (8.9)	2 (1.8)	1 (1.7)		

**Table 4:** Study habit scores (mean and SD) by academic programme, with significant group differences indicated using asterisks (*, *p* < 0.05). The significance of group differences was calculated using linear regression.

Study Habit Area	Overall (*n* = 576)	Applied Sciences (*n* = 56)	Engineering and Computer Sciences (*n* = 60)	Health Sciences (*n* = 324)	Humanities (*n* = 112)	Group Differences (*n* = 552)	*p*-Value
Surface score	26.39 (3.98)	25.04 (7.13)	27.73 (8.47)	26.45 (6.48)	25.91 (6.83)	F (3,548) = 1.67	0.171
Surface motive	11.61 (3.98)	11.36 (3.84)	12.95 (4.77)	11.38 (3.70)	11.59 (4.08)	F (3,548) = 2.77	0.041 *
Surface strategy	14.78 (3.80)	13.68 (3.94)	14.78 (4.58)	15.06 (3.65)	14.32 (3.59)	F (3,548) = 2.73	0.043 *
Deep score	30.79 (7.02)	31.52 (7.76)	31.92 (8.21)	30.40 (6.45)	30.85 (7.05)	F (3,548) = 1.09	0.353
Deep motive	15.99 (3.78)	16.18 (4.19)	16.43 (4.08)	15.97 (3.57)	15.72 (3.70)	F (3,548) = 0.53	0.662
Deep strategy	14.80 (3.87)	15.34 (4.22)	15.48 (4.52)	14.43 (3.60)	15.12 (3.83)	F (3,548) = 2.29	0.077

## Data Availability

The original contributions presented in this study are included in the article. Further inquiries can be directed to the corresponding author(s).

## Glossary

Neuroscience literacy: “understanding the brain and how it functions” [4]. Neuromyths: incorrect beliefs related to the brain and neuroscience are widespread [9].

## Abbreviations

GPA	Grade point average
SD	Standard deviation
R-SPQ-2F	Revised Two-factor Study Process Questionnaire
GAT	General aptitude score
VIF	Variance inflation factor
KAUH	King Abdulaziz University Hospital
